# Bayesian nonparametric estimation of EQ-5D utilities for United States using the existing United Kingdom data

**DOI:** 10.1186/s12955-017-0770-1

**Published:** 2017-10-06

**Authors:** Samer A. Kharroubi

**Affiliations:** Department of Nutrition and Food Sciences, Faculty of Agricultural and Food Sciences, American University of Beirut, Beirut, Lebanon

**Keywords:** Preference-based health measure, Non-parametric Bayesian methods, Time trade-off, EQ-5D

## Abstract

**Background:**

Valuations of health state descriptors such as EQ-5D or SF6D have been conducted in different countries. There is a scope to make use of the results in one country as informative priors to help with the analysis of a study in another, for this to enable better estimation to be obtained in the new country than analyzing its data separately.

**Methods:**

Data from 2 EQ-5D valuation studies were analyzed using the time trade-off technique, where values for 42 health states were devised from representative samples of the UK and US populations. A Bayesian non-parametric approach has been applied to predict the health utilities of the US population, where the UK results were used as informative priors in the model to improve their estimation.

**Results:**

The findings showed that employing additional information from the UK data helped in the production of US utility estimates much more precisely than would have been possible using the US study data alone.

**Conclusion:**

It is very plausible that this method would serve useful in countries where the conduction of large evaluation studies is not very feasible.

## Background

In the era of the preference based measures of health related quality of life (HRQoL), several multi-attribute health status classifications have been developed. Those tools include the EQ-5D [1], HUI2 and 3 [2, 3], AQoL [4], QWB [5] and the most recent SF-6D [6], in addition to condition specific classifications [7]. These tools allow the person to generate a description of their health state at a given point in time, in addition to the integration of an empirically deduced health state value that could be employed to estimate the quality adjusted life years (QALYs), a commonly used effectiveness measure in a specific form of cost-effectiveness analyses; cost-utility analysis [8].

Nowadays, the EQ-5D has become one of the most commonly used health preference tool to measure HRQoL, mainly in Europe, albeit gaining popularity in North America. One of the many cardinal handiness of the worldwide utilization of the EQ-5D is the possibility of employing the results of one country to improve those of another country, and for this to enable the generation of utility estimates of the second country much more precisely than would have been possible when implementing and analyzing the country’s data alone.

In a previous attempt to model health preference based data, Kharroubi et al. [9] have developed a non-parametric Bayesian method where the intrinsic characteristics of the individual health state valuation have been tackled, rendering the method more theoretically appropriate than the previously adopted conventional parametric models [6, 10, 11]. This method have been applied to the SF-6D UK health state preference data based on the standard gamble (SG) approach [12], and extended to address covariates [13]. Nevertheless, this work spread out extensively where it reached other countries and hence it has been adopted for the SF-6D HK and Japan valuation data [14, 15], in addition to other preference based measures such as the HUI2 UK [16]. Further, it has been extended to handle the joint US-UK EQ-5D with time trade off technique (TTO) [17, 18], and recently the joint UK-HK and UK-Japan SF-6D data set [19, 20].

Similarly to the benefits obtained from the worldwide application of the EQ-5D, the pivotal perk of the non-parametric Bayesian approach is the possibility to use the results generated in one country as informative priors in designing the model to be implemented in another country. To our knowledge, we haven’t come across any work exploring this potential benefit previously. It is likely that this kind of analysis (borrowing strength from existing countries’ valuations) will prove to permit much smaller studies than have hitherto been employed when developing valuations for new countries. This will be hugely important in countries without the same capacity to conduct large scale health state valuations.

The objectives of this research are (a) to develop a Bayesian statistical method to enable evidence from one country to serve as prior information for a study in another, and (b) to apply this method to the analysis of a valuation study for EQ-5D in US using the already existing UK data.

A brief description of both the UK and the US EQ-5D valuation studies and the data set adopted is provided in Methods section of this manuscript. Then, in Modelling section, we describe the Bayesian non-parametric model implemented in the work developed by Kharroubi et al. [9] with additional implemented novelties in methodological advances deemed necessary for the development of better estimates for the preference utility function. In Results section, we present the findings obtained using the modified Bayesian method applied on the US/UK EQ-5D data sets and compare it to the results generated by the original model of Kharroubi et al. [9]). Finally, we conclude in Discussion section by discussing our results while shedding light on how they could be implicated in future uses of the EQ-5D and modelling in this field.

## Methods

### The EQ-5D descriptive system

The EQ-5D has been developed by a group of researchers distributed over seven centers in five countries. It stands for the EuroQoL-5D or the European quality of life with 5 health dimensions being: mobility, self-care, usual activities, pain/discomfort, and anxiety/depression. Each dimension has 3 levels being no problem, moderate problem, and severe problem from level 1 to level 3 respectively; hence their combinations generate 243 different health states, and each state is described in the form of a five-digit code using the three levels. For example, 11,111 and 33,333 describe the best health state and worst state respectively. In addition, unconsciousness and immediate death have been added to the valuation process but not to the descriptive system in order to complete the process.

### The valuation survey and data set

For use in cost-effectiveness analysis of health technologies, we need to assign a value to each health state that represents its utility, with 1 corresponds to perfect health, 0 corresponds to being dead and negative scores corresponds to health states judged worse that being dead. Those utility indexes have been obtained through the survey developed by the UK Measurement and Valuation of Health (MVH) group at the University of York, using a variant of the visual analog scale (VAS) and the time trade off (TTO) techniques [21]. A representative sample of the UK general population were interviewed in their own homes using TTO and VAS, where they were asked to value 12 health states. A total of 42 health states of the EQ-5D (excluding full health) were valued in this way. The sample was selected using a stratified random sampling method to ensure a balance of very mild (e.g. 11,112, 11,211, 21,111 …), mild (e.g. 11,122, 11,113, 12,121 …), moderate (e.g. 13,212, 12,222, 22,222 …) and severe (e.g. 33,232, 32,223, 23,313 …) states. A detailed description of the study is provided elsewhere [10].

The EQ-5D valuation study in the US used the same states and valued them using the same methods, however shifting from the simple sampling design adopted in the UK study. In fact, the research group created a 4-stage cluster sampling design, focusing on 2 of the largest minority groups, the Hispanics and non-Hispanic [22]. The UK study interviewed a total of 3395 individuals with a response rate of 64%, while the US study interviewed a total of 4048 individuals with a response rate of 59.4%. However, after excluding respondents with incomplete or inconsistent responses, the usable valuation data ended up being obtained from 2997 and 3773 respondents from UK and US respectively, where both samples are representative of their populations on the sociodemographic and economic levels [10, 22].

Both the US and UK studies used the TTO method [10, 22] for eliciting health state value. Briefly, respondents were asked to bracket the number of years x (x ≤ 10) spent in full health which they value equivalent to 10 years spent in the state in question, for states considered better than death. Hence, the smaller the degree of indifference, the state is regarded as worse. For states considered worse than being dead,, respondents were asked to decide whether they preferred immediate death or spending (10-x) years in that state followed by x in full health. Then, in order to compensate for a shorter period in the state in question, more time is needed in the perfect health state 11,111 when x increases, thus indicating a worse health state. Afterwards, scores have been transformed based on the formula x/10, for states regarded as better than death, and -x/10, for states considered worse than death, in order to bound them on the scale −1 to 1, with x being the time spent in full health [10, 21].

Another difference between the UK and US studies is the allocation of the 42 health states across respondents. In the UK study, 41 health states (excluding 33,333) were spread over 4 groups based on the severity of the problem, where each individual was randomly assigned 11 states with varying severities (2 very mild, 3 mild, 3 moderate, and 3 severe) in addition to the 33,333 state. Whereas in the US study, respondents were randomized to obtain 1 of the 5 groups of predefined health states, where 4 groups were considered as the modelling sample and they each included: the worst state 33,333 in addition to 11 randomly selected health states (2 very mild states and 9 states selected from the remaining 36 states). As for the 5th group, the validation sample, it included the 33,333 health state and 11 health states randomly selected from the remaining 41 states. Further difference was that the interviews in UK were conducted in English, while in the US it has been done in either English or Spanish. Both valuation studies have been previously described in details [10, 22].

## Modelling

The preference based health state measure, EQ-5D, provides 243 possible health states, in the time when the empirical survey conducted could only gather a valuation for a small subset. Therefore, the purpose of modelling is to estimate health state utility values for all the EQ-5D states based on the 42 valued states. Parametric models with assumptions about the functional form have been implemented earlier. However, in their works, Kharroubi et al. [9] have developed a more realistic and flexible Bayesian non-parametric model to contrast the long used parametric method. In the next section, we will review the Bayesian non-parametric model created by Kharroubi et al. [9], which we shall refer to henceforth as the K-Model. This will form the basis of the development of the modified model in the following section. The latter will be referred to as the KM-Model.

### The K-Model

Kharroubi et al. [9] propose the following model

1 $$ {y}_{ij}=1-{\alpha}_j\left\{1-u\left({\mathbf{x}}_{ij}\right)\right\}+{\varepsilon}_{ij} $$

where *i* = 1,2,…,I_j_ and *j* = 1,2,…,*J, x*_*ij*_ is the *i*th health state valued by the respondent *j, y*_*ij*_ is the dependent variable representing the TTO valuation given by the respondent *j* for the specified health state, *α*_*j*_ is a random respondent residual term and *ε*_*ij*_ is a zero mean random error term. Kharroubi et al. [9] proposed the following distributions

$$ {\alpha}_j\sim LN\left({\mathbf{t}}_j^T\theta, {\tau}^2\right)\kern0.5em \mathrm{and}\kern0.5em {\varepsilon}_{ij}\sim N\left(0,{\upsilon}^2\right). $$

where **t**_*j*_ is the vector of covariates for respondent ***j***. Kharroubi et al. [9] next model the prior distribution for ***u***(**x**) as follows:

2 $$ u\left(\mathbf{x}\right)\sim N\left(\gamma +{\beta}^{\prime}\mathbf{x},{\sigma}^2\right) $$

Given Eq. (2), it is worth noting that **x** is a vector consisting of discrete levels on each of the five health dimensions. In addition, the mean function of (2) represents a belief that the predicted utility will be roughly linear and additive in its different dimensions, whereas the parametric model would have imposed the assumptions of linearity and additivity. In fact, the function in our model is free to vary around the mean based on its multivariate normal distribution, hence, taking unconditionally any functional form suggested by the data. Based on the latter difference, the model is described as non-parametric, rendering it more realistic and appropriate. For instance, if the data are strong, then they will over-rule the prior expectation. However, from a practical point of view, the data will be less strong, thus the prior model will smooth the empirical relationship suggested by the data towards the form suggested by the mean function of Eq. (2). More details on this are given in Kharroubi et al. [9].

Additionally, the values of *u*(**x**) and *u*(**x** ^′^) for two separate states **x** and **x** ^′^ have a correlation c(**x**, **x** ^′^) that decreases as the distance between **x** and **x** ^′^ increases, and is defined as

3 $$ \mathrm{c}\left(\mathbf{x},{\mathbf{x}}^{\prime}\right)=\exp \left\{-\sum {b}_d{\left({x}_d-{x}_d^{\prime}\right)}^2\right\} $$

where for d = 1,2,…,5, *x*_*d*_ and ${x}_d^{\prime }$ are the levels of dimension d in the health state x and x’ respectively, and *b*_*d*_ is a roughness parameter in the dimension d which controls how well the true utility function is expected to adhere to a linear form in a dimension d. This function has been employed to assert that if the states **x** and **x**’ are very similar (their levels are close in all dimensions, hence they might be adjacent), their utilities will be almost the same, thus the preference function varies smoothly with the shift in the health state. Kharroubi et al. [9] provide a more thorough explanation about this specific point.

Finally, it’s noted by Kharroubi et al. [9] that the population mean utility for a given health state x is defined as follows

4 $$ \overline{u}\left(\mathbf{x}\right)=1-E\left(\alpha \right)\left(1-u\left(\mathbf{x}\right)\right) $$

where *E*(*α*) is the mean value of α over the whole population. This will only be equal to 1 if the mean and median are the same, which is not generally the case. More details on the evaluation of *E*(*α*) are given in Kharroubi et al. [9].

### The KM-Model

In this section, we further elaborate the non-parametric model to include the existing UK results elicited from the K-Model as informative priors, in the aim to improve the accuracy of the predictions of the US population utility function.

As the case of the K-Model, the *i*th valuation provided by respondent *j* in the US study is modelled as follows

5 $$ {\tilde{\mathrm{y}}}_{ij}=1\hbox{-} {\tilde{\upalpha}}_j\left\{1\hbox{-} \tilde{\mathrm{u}}\left({\mathbf{x}}_{ij}\right)\right\}+{\upvarepsilon}_{ij} $$

where *ε*_*ij*_ is the error term having a distribution as *ε*_*ij*_~*N*(0, *ῦ* ^2^) and *ᾶ*_*j*_ is the random respondent effect. Similarly, the distribution of ***ᾶ***_***j***_ is ${\overset{\sim }{\alpha}}_j\sim LN\left({\mathbf{t}}_j^T\overset{\sim }{\theta },{\overset{\sim }{\tau}}^2\right)$.

We next assume $\tilde{\mathrm{u}}(x)$ to be the utility function of health state x evaluated in the US study, then based on Eq. (2), the prior distribution for $\tilde{\mathrm{u}}(x)$ is multivariate normal as well, with mean defined as

6 $$ E\left(\tilde{\mathrm{u}}\left(\mathbf{x}\right)\right)=E\left(u\left(\mathbf{x}\right)\right)+\tilde{\gamma}+{\tilde{\beta}}^{\prime}\mathbf{x} $$

and variance-covariance matrix

7 $$ \operatorname{cov}\left(\mathrm{u}\left(\mathbf{x}\right),\mathrm{u}\left({\mathbf{x}}^{\mathbf{\prime}}\right)\right)+{\tilde{\sigma}}^2\mathrm{c}\left(\mathbf{x},{\mathbf{x}}^{\mathbf{\prime}}\right) $$

where *E*(*u*(**x**)) and cov(u(**x**), u(**x** ^′^)) are the mean health utility of health state x and the variance-covariance matrix of *u*(**x**) and *u*(**x** ^′^) respectively, obtained from the analysis of the existing UK data, and c(**x**, **x** ^′^) is the correlation between $\tilde{\mathrm{u}}\left(\mathbf{x}\right)$ and $\tilde{\mathrm{u}}\left({\mathbf{x}}^{\mathbf{\prime}}\right)$ defined analogously to Eq. (3). Notice in general that, in addition to the advantages discussed in The K-Model section, the new modelling of the utility function $\tilde{\mathrm{u}}\left(\mathbf{x}\right)$ allows the existing data in one country to contribute substantial prior information to the analysis of the study in another country. Thus, the inclusion of *E*(*u*(**x**)) and cov(u(**x**), u(**x** ^′^)) in the mean function and the variance-covariance matrix of the prior distribution for $\tilde{\mathrm{u}}\left(\mathbf{x}\right)$ is more likely to produce estimation in the US much more precisely than would have been possible without it i.e. using the US data alone.

Finally, as noted by Kharroubi et al. [9], the population mean utility for a given health state x is defined as follows

8 $$ \overline{\tilde{\mathrm{u}}}\left(\mathbf{x}\right)=1-E\left(\tilde{\upalpha}\right)\left(1-\tilde{\mathrm{u}}\left(\mathbf{x}\right)\right) $$

where *E*(*ᾶ*) is the mean value of *ᾶ* over the whole US population. This will only be equal to 1 if the mean and median are the same, which is not generally the case. Therefore, the population’s mean health state utility of $\overline{\tilde{\mathrm{u}}}\left(\mathbf{x}\right)$ is not the same as the median health state utility $\tilde{\mathrm{u}}\left(\mathbf{x}\right)$.

General theory and full technical details of the new Bayesian statistical model in this article are given in Kharroubi [23]. Programs to undertake the Bayesian approach were written in Matlab. We will be pleased to supply the Matlab codes on request. However, these codes are not general and the user will need to modify them for his/her own purposes.

## Results

We now apply the KM-model to the analysis of a valuation study for EQ-5D in the US using the already existing UK data. The posterior distribution of the UK utility function will be used as a prior distribution to analyze the study in the US. This will be compared to the analysis of the US data alone using the original K-model. The two models are compared in terms of their predictive ability, including plots of predicted to actual values, calculations of the root mean squared error (RMSE) and plots of the standardized residuals and the Bland-Altman agreement plots. These assessments are undertaken within the full estimation sample and in an out of sample random selection of 3 states by re-estimating the models using data sets excluding these 3 states.

**Fig. 1:**
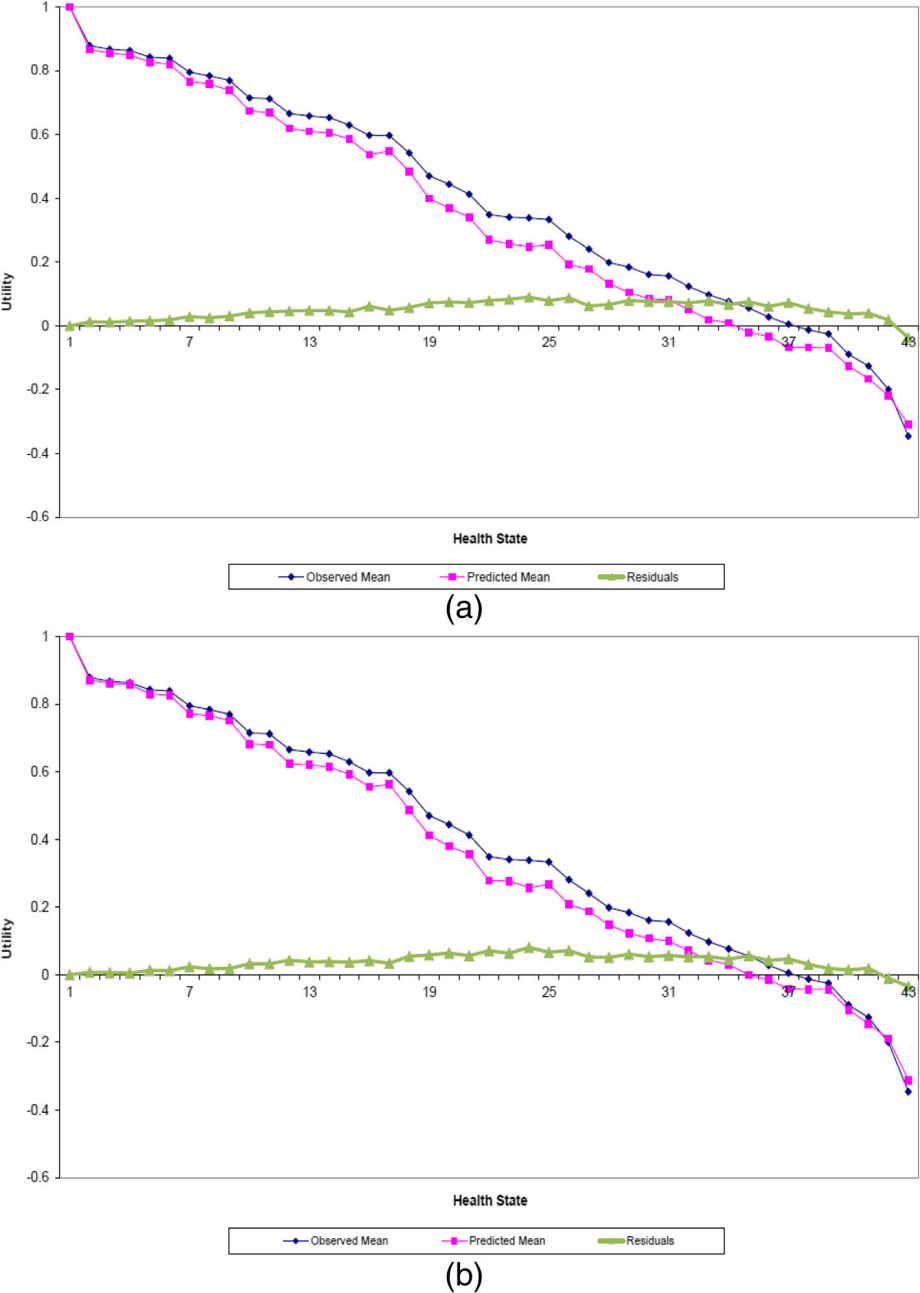
Sample mean and predicted health states valuations for **a** the K-Model and **b** the K-Model

**Fig. 2:**
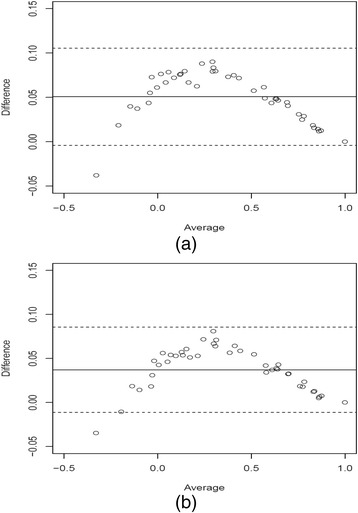
Bland-Altman agreement plots for **a** the K-Model and **b** the K-Model

The two models are compared in terms of their predictive ability in Figs. 1 and 2, where the predicted and actual mean values for the 42 health states valued in the survey together with the full health, ordered according to the predicted values. Figure 1a shows the US predicted mean health state valuations (squared line) using the K-Model, along with the actual mean health state valuations (diamond marked line), in addition to the errors computed by calculating the difference between the two valuations (triangles marked line). Whereas Fig. 1b reflects the results obtained using KM-Model, corresponding to the US data having UK results as informative priors. When comparing the plots, it is clear that the KM-Model predicts the data quite well, and better than the K-Model for all health states. In particular, although the KM-Model provides marginally better predictions for moderate health states, it produces quite well predictions for the mild (health states 2–9 on the graph) and severe (health states 39–43 on the graph) health states. Moreover, the plots reflect a larger difference between the valuations for the K-Model, indicating that the KM-Model is less prone to systematic bias.

For an improved quantification of the gains in terms of bias, Fig. 2a shows the Bland-Altman agreement plot [24], where the difference scores of the predicted and actual mean health state valuations are plotted against the average scores of the two valuations for the K-Model. The solid line represents the average bias (or the average of the differences) and the dotted lines are the 95% limits of agreement. Figure 2b presents the corresponding agreement plot for the KM-model. The plots suggest that the KM-Model shows a better agreement since the width of the 95% limits of agreement is equal to 0.096 (0.085 – (−0.011), which is narrower than that of the K-Model, which is equal to 0.109 (0.105 – (−0.004)). In addition, the difference in average bias between the KM-Model and K-Model is 0.037 and 0.051 respectively, where the difference for the KM-Model is smaller. Similarly, the standard deviation of the differences for the KM-Model is also smaller than that of the K-Model with the respective values of 0.024 and 0.028 respectively, thus justifying the large variations of the differences in Fig. 2a. In contrast, it is clear from Fig. 2b that the KM-Model differences are well validated.

The inferences for the mean health state utility values of the 42 states valued in the valuation survey are shown in Table 1. For each state, the predicted mean, standard deviation and corresponding 95% credible interval for the population mean health state utility from both models, in addition to the results for the population mean health state utility from the UK study that were used as informative priors in the KM-Model. Notice that some of these states (those marked with an asterisk) were randomly selected from the 200 remaining EQ-5D health states (excluding full health) that were not used in the experimental study and so estimates are being derived from the fitted model. As can be seen, throughout the 42 states (omitting the perfect health), the KM-Model proved to serve as a better predictive tool with a root mean square error (RMSE) of 0.044 versus 0.058 for the K-Model.

**Table 1:** Posterior inferences for utilities of the 43 health states together with 10 new health states not valued in the empirical survey

State X	Observed mean	UK results			K-Model			KM-Model		
		Posterior mean	Posterior SD	95% CI	Posterior mean	Posterior SD	95% CI	Posterior mean	Posterior SD	95% CI
11111	1	1	0		1	0		1	0	
11112	0.8385	0.8259	0.0091	(0.8081,0.8437)	0.8199	0.0089	(0.8025,0.8373)	0.8263	0.0085	(0.8096,0.8430)
11113	0.5422	0.4107	0.012	(0.3872,0.4342)	0.4848	0.0139	(0.4576,0.5120)	0.4875	0.0128	(0.4624,0.5126)
11121	0.8632	0.8494	0.0086	(0.8325,0.8663)	0.849	0.0102	(0.8290,0.8690)	0.8583	0.0095	(0.8397,0.8769)
11122	0.7693	0.7187	0.012	(0.6952,0.7422)	0.7386	0.0125	(0.7141,0.7631)	0.7509	0.0118	(0.7278,0.7740)
11131	0.3485	0.2238	0.013	(0.1983,0.2493)	0.2691	0.0148	(0.2401,0.2981)	0.2776	0.0142	(0.2498,0.3054)
11133	0.1988	0.0107	0.0131	(−0.0150,0.0364)	0.1321	0.0129	(0.1068,0.1574)	0.1476	0.0123	(0.1235,0.1717)
11211	0.8672	0.8704	0.0091	(0.8526,0.8882)	0.8556	0.0088	(0.8384,0.8728)	0.8609	0.0086	(0.8440,0.8778)
11312	0.63	0.5499	0.0123	(0.5258,0.5740)	0.5863	0.0125	(0.5618,0.6108)	0.5931	0.0126	(0.5684,0.6178)
11331^a^		0.2154	0.1522	(−0.0829,0.5137)	0.3027	0.1474	(0.0138,0.5916)	0.3275	0.1774	(−0.0202,0.6752)
12111	0.8429	0.8303	0.0089	(0.8129,0.8477)	0.8272	0.0107	(0.8062,0.8482)	0.8301	0.0098	(0.8109,0.8493)
12121	0.7837	0.7421	0.0121	(0.7184,0.7658)	0.7587	0.0123	(0.7346,0.7828)	0.7659	0.0115	(0.7434,0.7884)
12211	0.7951	0.763	0.012	(0.7395,0.7865)	0.7662	0.013	(0.7407,0.7917)	0.7716	0.0115	(0.7491,0.7941)
12222	0.6533	0.5467	0.0118	(0.5236,0.5698)	0.6052	0.0111	(0.5834,0.6270)	0.6145	0.0101	(0.5947,0.6343)
12223	0.4441	0.2411	0.0118	(0.2180,0.2642)	0.3693	0.0149	(0.3401,0.3985)	0.3799	0.0133	(0.3538,0.4060)
12322^a^		0.3008	0.1012	(0.1024,0.4992)	0.3771	0.0993	(0.1825,0.5717)	0.3888	0.1213	(0.1511,0.6265)
13212	0.4704	0.3958	0.0123	(0.3717,0.4199)	0.3988	0.0148	(0.3698,0.4278)	0.4119	0.0124	(0.3876,0.4362)
13311	0.4131	0.3549	0.0128	(0.3298,0.3800)	0.3402	0.0119	(0.3169,0.3635)	0.3566	0.0113	(0.3345,0.3787)
13313^a^		0.1381	0.1172	(−0.0916,0.3678)	0.2134	0.115	(−0.0120,0.4388)	0.2302	0.1376	(−0.0395,0.4999)
13332	−0.0131	−0.1304	0.0144	(−0.1586,−0.1022)	−0.0679	0.0171	(−0.1014,−0.0344)	−0.0438	0.0157	(−0.0746,0.0130)
21111	0.8788	0.8788	0.0098	(0.8596,0.8980)	0.8662	0.0088	(0.8490,0.8834)	0.8713	0.0084	(0.8548,0.8878)
21133	0.1612	−0.0105	0.014	(−0.0379,0.0169)	0.0851	0.0162	(0.0533,0.1169)	0.1077	0.0145	(0.0793,0.1361)
21222	0.6587	0.5496	0.0115	(0.5271,0.5721)	0.6104	0.0111	(0.5886,0.6322)	0.621	0.0101	(0.6012,0.6408)
21232	0.3382	0.1008	0.0125	(0.0763,0.1253)	0.248	0.0153	(0.2180,0.2780)	0.2574	0.0132	(0.2315,0.2833)
21311^a^		0.5801	0.117	(0.3508,0.8094)	0.5696	0.1144	(0.3454,0.7938)	0.5899	0.1322	(0.3308,0.8490)
21312	0.5972	0.5388	0.0129	(0.5135,0.5641)	0.5483	0.0129	(0.5230,0.5736)	0.5632	0.0124	(0.5389,0.5875)
21323	0.3402	0.1687	0.0131	(0.1430,0.1944)	0.2569	0.0155	(0.2265,0.2873)	0.2765	0.014	(0.2491,0.3039)
22112	0.7129	0.6642	0.012	(0.6407,0.6877)	0.6685	0.0122	(0.6446,0.6924)	0.6805	0.0119	(0.6572,0.7038)
22121	0.7154	0.6361	0.0118	(0.6130,0.6592)	0.6749	0.0112	(0.6529,0.6969)	0.6828	0.0101	(0.6630,0.7026)
22122	0.6664	0.5415	0.0116	(0.5188,0.5642)	0.6198	0.0127	(0.5949,0.6447)	0.6234	0.0119	(0.6001,0.6467)
22222	0.5979	0.4985	0.0121	(0.4748,0.5222)	0.5365	0.0128	(0.5114,0.5616)	0.5561	0.0122	(0.5322,0.5800)
22232^a^		0.0989	0.0682	(−0.0348,0.2326)	0.1903	0.0671	(0.0588,0.3218)	0.2098	0.081	(0.0510,0.3686)
22233	0.1236	−0.088	0.0141	(−0.1156,-0.0604)	0.0515	0.0153	(0.0215,0.0815)	0.0707	0.0145	(0.0423,0.0991)
22323	0.2804	0.0743	0.0129	(0.0490,0.0996)	0.1926	0.0157	(0.1618,0.2234)	0.2089	0.0136	(0.1822,0.2356)
22331	0.1844	0.011	0.0139	(−0.0162,0.0382)	0.105	0.013	(0.0795,0.1305)	0.1236	0.0124	(0.0993,0.1479)
23223^a^		−0.0038	0.1121	(−0.2235,0.2159)	0.0742	0.1101	(−0.1416,0.2900)	0.0928	0.1319	(−0.1657,0.3513)
23232	0.0973	−0.0305	0.0138	(−0.0575,-0.0035)	0.0191	0.0163	(−0.0128,0.0510)	0.0434	0.0144	(0.0152,0.0716)
23313	0.1569	−0.0017	0.0137	(−0.0286,0.0252)	0.0814	0.0134	(0.0551,0.1077)	0.0997	0.0129	(0.0744,0.1250)
23321	0.3331	0.1555	0.013	(0.1300,0.1810)	0.2539	0.0126	(0.2292,0.2786)	0.2666	0.012	(0.2431,0.2901)
31221^a^		0.2783	0.1307	(0.0221,0.5345)	0.3016	0.128	(0.0507,0.5525)	0.3127	0.1517	(0.0154,0.6100)
32211	0.2408	0.1779	0.0124	(0.1536,0.2022)	0.1783	0.016	(0.1469,0.2097)	0.188	0.0144	(0.1598,0.2162)
32212^a^		0.1528	0.0902	(−0.0240,0.3296)	0.1601	0.0889	(−0.0141,0.3343)	0.1813	0.1056	(−0.0257,0.3883)
32223	0.0558	−0.0991	0.0142	(−0.1269,−0.0713)	−0.0203	0.0158	(−0.0513,0.0107)	−0.0003	0.0158	(−0.0313,0.0307)
32232	0.0051	−0.1201	0.0136	(−0.1468,−0.0934)	−0.0677	0.0165	(−0.1000,−0.0354)	−0.0419	0.0158	(−0.0729,−0.0109)
32313	0.0278	−0.1005	0.0145	(−0.1289,−0.0721)	−0.0331	0.0152	(−0.0629,−0.0033)	−0.0148	0.0151	(−0.0444,0.0148)
32331	−0.0895	−0.1838	0.0152	(−0.2136,−0.1540)	−0.1269	0.0169	(−0.1600,−0.0938)	−0.1038	0.0151	(−0.1334,−0.0742)
33133^a^		−0.3087	0.1549	(−0.6123,−0.0051)	−0.2178	0.1522	(−0.5161,0.0805)	−0.2129	0.1788	(−0.5633,0.1375)
33212	0.0765	0.0163	0.0131	(−0.0094,0.0420)	0.0097	0.017	(−0.0236,0.0430)	0.0303	0.0146	(0.0017,0.0589)
33232	−0.1263	−0.2231	0.0136	(−0.2498,−0.1964)	−0.1662	0.0169	(−0.1993,−0.1331)	−0.1449	0.0159	(−0.1761,−0.1137)
33321	−0.0254	−0.073	0.0142	(−0.1008,−0.0452)	−0.0692	0.0144	(−0.0974,−0.0410)	−0.0436	0.0132	(−0.0695,−0.0177)
33323	−0.1999	−0.2559	0.0142	(−0.2837,−0.2281)	−0.2184	0.0172	(−0.2521,−0.1847)	−0.1894	0.0155	(−0.2198,−0.1590)
33332^a^		−0.3003	0.0958	(−0.4881,−0.1125)	−0.2564	0.0942	(−0.4410,−0.0718)	−0.2282	0.1082	(−0.4403,−0.0161)
33333	−0.346	−0.3599	0.0102	(−0.3799,−0.3399)	−0.3082	0.0121	(−0.3319,−0.2845)	−0.3114	0.0116	(−0.3341,−0.2887)

*SD* Standard Deviation, *CI* Credible Interval; ^a^represent the 10 health states not valued in the empirical survey

Other significant differences between the models are clearly reflected in Table 1. For instance, the pits state has a predictive utility of −0.3082 from the K-Model and −0.3114 from the KM-Model, whereas the observed value is −0.346. Moreover, the standard deviations of the KM-Model are smaller since it employs the UK results as priors, hence the better estimation. Other spotted performance differences between the models include monotonicity. In fact, out of the 243 adjacent health state pairs, non-monotonicity is observed by 20% of the cases in the K-Model, while the rate is 10% in the KM-Model.

**Fig. 3:**
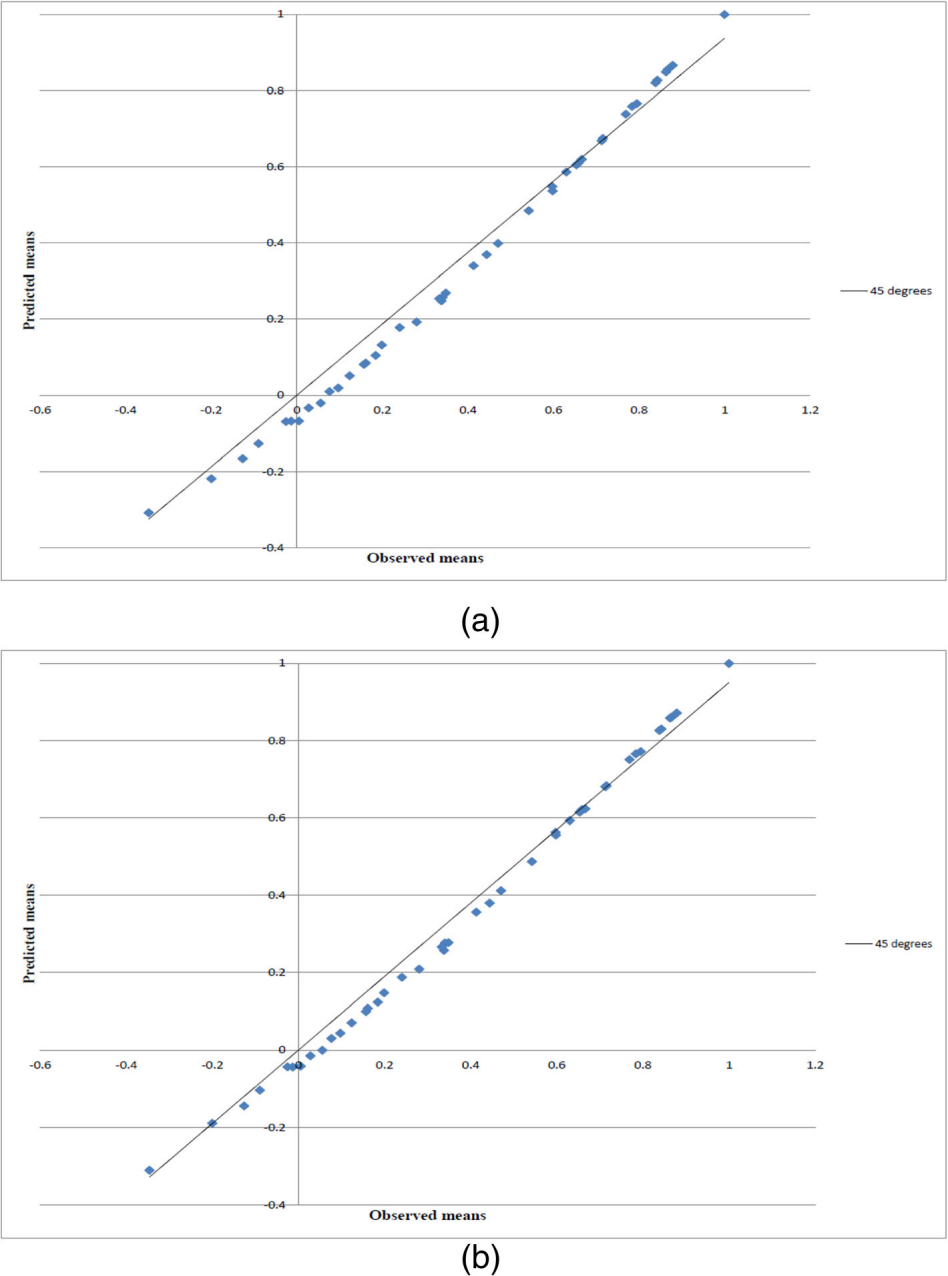
Sample mean and predicted health states valuations for **a** the K-Model and **b** the K-Model

A clearer representation of the differences is reflected in Fig. 3, which shows the predicted values using the K-Model and KM-Model against observed mean values of the 42 health states, in addition to the perfect predictions indicated by a 45-degree line of unity (solid line). In theory, we would expect the predicted values from the two models to lie roughly on the perfect predictions line. Despite the good validation of the models by their predictive performance, Fig. 3b shows the predictions from the KM-model to be closer to the theoretical line, as opposed to Fig. 3a, which shows a larger scatter of the deviating points from the solid line. Therefore, we could stress the fact that the KM-Model produces better predictions.

**Fig. 4:**
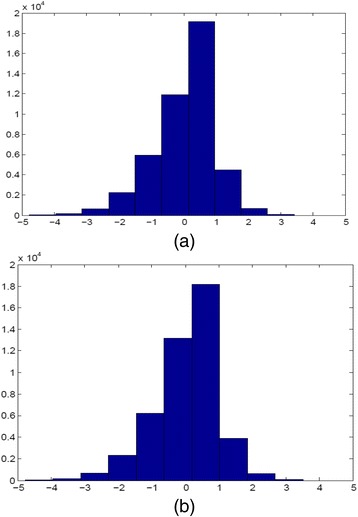
Standardized residuals for each of the 45,276 individual health state valuations: **a** for the K-Model and **b** for the K-Model

Another aspect to verify the validity of the models is through plotting histograms of standardized residuals. Thus, Fig. 4a and b present histograms for the standardized residuals across all 45,276 valuations for the K-Model and KM-Model respectively. Theoretically, we would expect these to follow a *N*(0,1). In practice, the theory is generally supported by both figures, although there a slight evidence of skewness. However, the skewness shown in both Figures is modest; hence we feel that the analyses of both models are not seriously invalidated.

To this end, we perform an out-of-sample leave-one-out prediction at the level of health states. This is done by sequentially removing three of the 42 observed health states from both sets of data, fitting the model to the remaining 41 (using the UK 41 as informative priors), then making a prediction for the left out state in the US data. Table 2 displays the resulting out-of-sample predictions from both models, along with their observed mean values from the valuation study. Results showed that the KM-Model overtakes the K-Model in its predictive performance, with a RMSE of 0.0274 for the KM-model compared to 0.0358 for the K-Model. It is worth noting finally that the posterior standard deviations in Table 2 are larger than those in Table 1 since this analysis was conducted on an out-of-sample data, whereas previously, we were predicting pre-estimated data.

**Table 2:** Prediction of reserved data means

Missing state	Observed mean	K-Model			KM-Model		
		Posterior mean	Posterior SD	95% CI	Posterior mean	Posterior SD	95% CI
12121	0.7837	0.7426	0.0866	(0.5729,0.9123)	0.7515	0.0854	(0.5841,0.9189)
22331	0.1844	0.1381	0.1117	(−0.0808,0.3570)	0.1496	0.1109	(−0.0678,0.3670)
33232	−0.1263	−0.1218	0.0977	(−0.3133, 0.0697)	−0.1245	0.0964	(−0.3134,0.0644)

*SD* Standard Deviation**,** *CI* Credible Interval

## Discussion

In this paper we have developed a Bayesian statistical method for estimating the utility values of health states defined by the EQ-5D generic descriptive system, in order to generate QALYs and hence to conduct cost utility analysis of health care interventions. The new method enables evidence from one country to serve as prior information for a study in another. We have also applied this method to the analysis of a valuation study for EQ- 5D in United States using the already existing United Kingdom data. The methodology builds on a successful Bayesian nonparametric modelling of the UK EQ-5D valuation data. The posterior distribution of the UK utility function was used as a prior distribution to analyze the new study in the US.

We have shown that the new modelling of the utility function allowed the UK data to contribute substantial prior information to the analysis of the US study. As a result, the US utilities for the 42 EQ-5D health states were estimated much more precisely than would have been possible using the US study data alone, yet respect the inherent monotonicity of the underlying utility measure even further. Careful model checking, including prediction of left-out data, confirm that the new KM-model fits well and better than that of the K-model.

The novel part of the analysis was to make use of experience in one country to help with the analysis of a study in another. There is also a scope to make use of this to help with the design of a study in another, and for this to enable a smaller sample to be used in the new country. The choice of new health states to be valued in a follow-on study was made to provide information primarily about parts of the EQ-5D descriptive space of 243 health states that were less estimated from the US study alone and the UK evidence. Work is in progress on demonstrating this idea in the context of a smaller country.

In the analysis presented here we have data on two countries that are culturally similar (UK and US). The results suggest that drawing extra information from the UK produces better estimation of the US utilities than using the US data alone. The next thing would be worthwhile to explore is the use of our model when we have data on two countries that are sufficiently different. Work is also in progress on exploring whether using the UK data might help with the design and analysis of a valuation study for SF-6D in Hong Kong.

Health technology assessment is an international endeavor, with pharmaceuticals clinical trials are being conducted in different countries. The World Health Organization undertakes cost-effectiveness analysis of interventions across national boundaries. As more statutory funding bodies around the world demand cost-effectiveness assessments, effectiveness analysis will become more international with syntheses of data across countries. A key element in this will be the valuation of health states in order to calculate QALYs. Thus, precise estimation of health state utility values is an important component of this. For instance, the K-Model estimates the health utility for state 33,323 to be −0.2184, whereas the KM-Model achieves −0.1894, and so the difference in utility estimates is almost 0.03. This could result in an increase in QALYs from a treatment that extends life by one year of 0.5 to 0.53, which for a treatment costing £10,000 would reduce the cost per QALY from £20,000 to £18,867 and bring it below the cost effectiveness threshold used by NICE. Heijink et al. [25] have reported similar impact of different valuation functions on QALYs.

The modified model presented here is also applicable to other preference-based measures such as SF-6D and HUI, as well as to more condition specific preference-based measures. Ongoing work is on the application to SF-6D measure. Matlab code for undertaking the KM-model and K-model is available upon request.

## Conclusion

In conclusion, the new Bayesian statistical method is a powerful technique that might be applied to design and analyze health-related quality of life utility valuation studies for wide range of health state descriptive systems when data already exist in another country. It is likely that this kind of analysis (borrowing strength from existing countries’ valuations) will prove to permit much smaller studies than have hitherto been employed when developing valuations for new countries. The implications of these results will be hugely important in countries without the same capacity to conduct large scale health state valuations.

## Abbreviations

AQoL: Assessment of Quality of Life
EQ-5D: The EuroQol
HRQoL: Health-related quality of life
HUI: Health Utilities Index
LN(.,.): Log-Normal distribution
MLE: Maximum likelihood estimator
MVH: Measurement and Valuation of Health
N(.,.): Normal distribution
QALYs: Quality adjusted life years
QWB: Quality of well-being
RMSE: Root mean square error
SD: Standard deviation
SF-6D: Short form 6 dimensions health survey
SG: Standard gamble
TTO: Time trade-off
UK: United Kingdom
VAS: Visual analogue scale
